# Modalities of Vitamin D Administration to Preterm Infants: Impact on 25 OH Vitamin D Levels

**DOI:** 10.1111/apa.70422

**Published:** 2025-12-17

**Authors:** Sophie Laborie, Manon Breniaux, Justine Bacchetta, Marine Butin

**Affiliations:** ^1^ Department of Neonatal Intensive Care Hôpital Femme Mère Enfant, Hospices Civils de Lyon Bron France; ^2^ Pharmacy Hôpital Femme Mère Enfant, Hospices Civils de Lyon Bron France; ^3^ Faculté de Médecine Lyon Est Université Claude Bernard Lyon 1 Lyon France; ^4^ Service de Néphrologie, Rhumatologie et Dermatologie Pédiatriques, Centre de Référence des Maladies Rares du Calcium et du Phosphore, Hôpital Femme Mère Enfant, Hospices Civils de Lyon Bron France; ^5^ INSERM 1033, Prévention des Maladies Osseuses Lyon France; ^6^ INSERM U1111, CNRS UMR5308, Centre International de Recherche en Infectiologie, Ecole Normale Supérieure de Lyon Université Claude Bernard Lyon 1 Lyon France

**Abbreviations**
25(OH)D: 25‐hydroxyvitamin D
NICUs: Neonatal Intensive Care Units

1

Among preterm infants, low serum 25‐hydroxyvitamin D (25(OH)D) levels are associated with increased morbidities, including bronchopulmonary dysplasia, whereas excessive levels may lead to hypercalcemia, hypercalciuria, and nephrocalcinosis [1].

For vitamin D intakes, U.S. recommendations suggest 200–400 IU/day; European guidelines advise 400–700 IU/kg/day up to 1000 IU/day, monitoring is suggested. In France, 2022 guidelines recommend 600–1000 IU/day of enteral vitamin D for extremely and very preterm infants, with monthly monitoring of 25(OH)D and a target of 50–120 nmol/L [2].

Three enteral formulations are available in France: Uvesterol ADEC (Crinex, Gentilly, France): aqueous multivitamin with ergocalciferol, 1000 IU/0.3 mL, Adrigyl (Crinex, Gentilly, France): cholecalciferol, lipidic, 333 IU/drop, Zyma D (Viatris Santé, Lyon, France): cholecalciferol, lipidic, 300 IU/drop. Despite frequent NICU use of alfacalcidol or calcitriol, we found no published guidelines recommending them for preterm infants [3].

Vitamin D, being lipophilic, can sorb to plastics, with losses influenced by formulation, excipients, tubing materials, and administration technique [4]. We hypothesised that variability in 25(OH)D levels partly reflects differences in dosing practices and delivery methods.

A national survey was conducted in 2024 across 54 neonatal French neonatal intensive care units (NICUs). Data collected included parenteral and enteral supplementation practices, monitoring strategies, dose adjustments, incidence of deficiency or excess [2], and use of vitamin D analogs were recorded.

Results were descriptive. Fisher's exact test assessed associations between practices and abnormalities (*p* < 0.05).

Among the 54 centers, 41 (76%) responded: 24/41 (59%) routinely measured 25(OH)D levels, 19/41 (46%) adjusted native vitamin D doses, 30/41 (73%) used alfacalcidol, and only 1/41 (2%) used calcitriol.

Parenteral supplementation ranged from 33 to 500 IU/kg/day or 32–400 IU/day, with medians of 120 IU/kg/day and 110 IU/day.

Enteral supplementation practices varied: 10/41 NICUs (24%) used cholecalciferol, 31/41 (76%) used ergocalciferol, and doses were 1000 IU/day (24/41, 56%), 660 IU/day (6/41, 15%), 600 IU/day (5/41, 12%), or variable (6/41, 15%).

The relationship between enteral dosing, administration methods, and the occurrence of deficiency or excess is shown in Figure 1.

**FIGURE 1:**
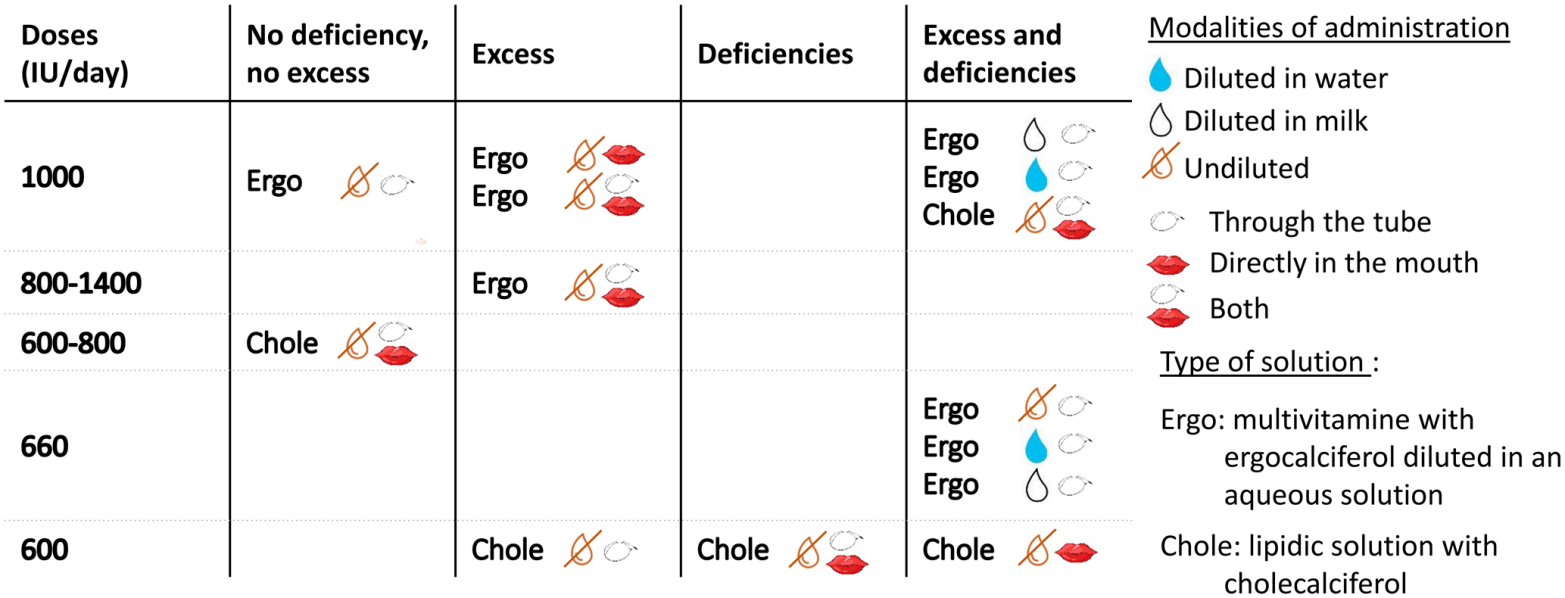
Report on deficiencies and/or excesses of 25(OH)D levels in NICUs, based on the dose and administration method of native vitamin D.

Vitamin D excess occurred in 3 NICUs despite low total parenteral (44–55 IU/day) and enteral (600–660 IU/day) intakes. One NICU using 160 IU/kg/day parenteral followed by 1000 IU/day of enteral cholecalciferol reported both deficiency and excess.

NICUs using 600–660 IU/day reported more frequent deficiencies than those giving 1000 IU/day (7/8 vs. 4/10; *p* = 0.053).

NICUs administering undiluted formulations containing ergocalciferol at 1000 IU/day had significantly fewer deficiencies than those using diluted formulations (0/5 vs. 3/4; *p* = 0.048).

Among the NICUs using alfacalcidol, 5/30 (17%) used it more than once a month. Indications included severe hypocalcemia (29/30, 97%), metabolic bone disease (7/30, 23%), and kidney failure (1/30, 3%). Treated infants were extremely (10/30, 30%), very (15/30, 50%), and moderately preterm infants (25/30, 83%). The dosages ranged from 0.1 μg/day to 2 μg/kg/day. The pre‐treatment labs included calcium and phosphate (29/30, 97%), PTH (15/30, 50%), 25(OH)D (14/30, 47%), ALP (10/30, 33%), and 1,25(OH)₂D (6/30, 20%). Treatment was mostly short (< 10 days in 90% (27/30)); it never exceeded 1 month. Dose adjustments were mainly based on circulating calcium (25/30.83%).

Parenteral vitamin D doses were often below recommendations due to the use of multivitamin preparations formulated for older children with low vitamin D content.

Despite low parenteral doses, some NICUs reported excess, possibly due to high enteral supplementation or maternal prenatal vitamin D intake (100 000 IU recommended during the seventh month).

On the other hand, a NICU using high‐dose cholecalciferol reported deficiencies, likely related to administration losses. Albinsson et al. [4] showed substantial losses of vitamin D in syringes and feeding tubes due to adsorption to plastic surfaces or dead space. Losses depend on vitamin lipophilicity, pKa, steric hindrance, excipients, flow rates, tubing length, temperature, and plastic composition.

Multivitamin solutions containing vitamin C improved vitamin D recovery, possibly by reducing hydrophobic interactions [4]. Dilution may reduce adsorption but could compromise stability under light or heat, although degradation is generally slow unless reactive metals are present.

Although all NICUs using cholecalciferol administered it undiluted, deficiencies still occurred even at high doses and via oral delivery. Practical factors—such as challenges in swallowing drops, retention in syringe dead space, or adhesion to tubing—likely limit effective delivery. Sublingual absorption remains unvalidated in preterm populations.

This nationwide survey highlights heterogeneity in vitamin D practices across French NICUs, consistent with findings from the U.S. [5] and England [3]. Variability concerns not only dosing and monitoring frequency but also formulation type and technical aspects of administration. Administration of 1000 IU/day of ergocalciferol in a multivitamin formulation appeared more effective in preventing deficiency than lipid‐based cholecalciferol, though not without risk of excess. Practices involving cholecalciferol showed greater variability, likely due to sorption‐related losses. These findings indicate that clinical outcomes depend not only on prescribed dose but also on formulation, concentration, and delivery technique.

Trials and international guidelines should address not only dosing and monitoring but also physicochemical and technical factors influencing bioavailability.

In the meantime, regular monitoring of 25(OH)D remains essential to avoid both deficiency and excess. In addition, the widespread but inconsistent use of vitamin D analogs calls for controlled trials to determine safety, efficacy, and optimal dosing in preterm infants.

## Author Contributions

**Sophie Laborie:** conceptualization, methodology, data curation, investigation, validation, formal analysis, writing – original draft, software. **Manon Breniaux:** validation, writing – review and editing, resources. **Justine Bacchetta:** conceptualization, validation, supervision, writing – review and editing, resources. **Marine Butin:** validation, visualization, writing – review and editing, supervision.

## Funding

The authors have nothing to report.

## Conflicts of Interest

The authors declare no conflicts of interest.

## Data Availability

The data that support the findings of this study are available from the corresponding author upon reasonable request.
